# The UniProt website API: facilitating programmatic access to protein knowledge

**DOI:** 10.1093/nar/gkaf394

**Published:** 2025-05-07

**Authors:** Shadab Ahmad, Leonardo Jose da Costa Gonzales, Emily H Bowler-Barnett, Daniel L Rice, Minjoon Kim, Supun Wijerathne, Aurélien Luciani, Swaathi Kandasaamy, Jie Luo, Xavier Watkins, Edd Turner, Maria J Martin, the UniProt Consortium, Alex Bateman, Maria-Jesus Martin, Sandra Orchard, Michele Magrane, Shiqi Ye, Aduragbemi Adesina, Shadab Ahmad, Emily H Bowler-Barnett, David Carpentier, Paul Denny, Jun Fan, Leonardo Jose da Costa Gonzales, Abdulrahman Hussein, Alexandr Ignatchenko, Giuseppe Insana, Rizwan Ishtiaq, Vishal Joshi, Dushyanth Jyothi, Swaathi Kandasaamy, Antonia Lock, Aurelien Luciani, Jie Luo, Yvonne Lussi, Juan Sebastian Martinez Marin, Pedro Raposo, Daniel L Rice, James Stephenson, Prabhat Totoo, Nadya Urakova, Preethi Vasudev, Supun Wijerathne, Khawaja Talal Ibrahim, Minjoon Kim, Conny Wing-Heng Yu, Alan J Bridge, Lucila Aimo, Ghislaine Argoud-Puy, Andrea H Auchincloss, Kristian B Axelsen, Parit Bansal, Delphine Baratin, Teresa M Batista Neto, Marie-Claude Blatter, Jerven T Bolleman, Emmanuel Boutet, Lionel Breuza, Cristina Casals-Casas, Kamal Chikh Echioukh, Elisabeth Coudert, Beatrice Cuche, Edouard de Castro, Anne Estreicher, Maria L Famiglietti, Marc Feuermann, Elisabeth Gasteiger, Pascale Gaudet, Sebastien Gehant, Vivienne Gerritsen, Arnaud Gos, Nadine Gruaz, Chantal Hulo, Nevila Hyka-Nouspikel, Florence Jungo, Arnaud Kerhornou, Philippe Le Mercier, Damien Lieberherr, Patrick Masson, Anne Morgat, Salvo Paesano, Ivo Pedruzzi, Sandrine Pilbout, Lucille Pourcel, Sylvain Poux, Monica Pozzato, Manuela Pruess, Nicole Redaschi, Catherine Rivoire, Christian J A Sigrist, Karin Sonesson, Shyamala Sundaram, Anastasia Sveshnikova, Cathy H Wu, Chuming Chen, Hongzhan Huang, Kati Laiho, Minna Lehvaslaiho, Peter McGarvey, Darren A Natale, Karen Ross, C R Vinayaka, Yuqi Wang, Raja Mazumber, Vijay Shanker, Jian Zhang

**Affiliations:** European Molecular Biology Laboratory, European Bioinformatics Institute (EMBL-EBI), Wellcome Genome Campus, Hinxton CB10 1SD, United Kingdom; European Molecular Biology Laboratory, European Bioinformatics Institute (EMBL-EBI), Wellcome Genome Campus, Hinxton CB10 1SD, United Kingdom; European Molecular Biology Laboratory, European Bioinformatics Institute (EMBL-EBI), Wellcome Genome Campus, Hinxton CB10 1SD, United Kingdom; European Molecular Biology Laboratory, European Bioinformatics Institute (EMBL-EBI), Wellcome Genome Campus, Hinxton CB10 1SD, United Kingdom; European Molecular Biology Laboratory, European Bioinformatics Institute (EMBL-EBI), Wellcome Genome Campus, Hinxton CB10 1SD, United Kingdom; European Molecular Biology Laboratory, European Bioinformatics Institute (EMBL-EBI), Wellcome Genome Campus, Hinxton CB10 1SD, United Kingdom; European Molecular Biology Laboratory, European Bioinformatics Institute (EMBL-EBI), Wellcome Genome Campus, Hinxton CB10 1SD, United Kingdom; European Molecular Biology Laboratory, European Bioinformatics Institute (EMBL-EBI), Wellcome Genome Campus, Hinxton CB10 1SD, United Kingdom; European Molecular Biology Laboratory, European Bioinformatics Institute (EMBL-EBI), Wellcome Genome Campus, Hinxton CB10 1SD, United Kingdom; European Molecular Biology Laboratory, European Bioinformatics Institute (EMBL-EBI), Wellcome Genome Campus, Hinxton CB10 1SD, United Kingdom; European Molecular Biology Laboratory, European Bioinformatics Institute (EMBL-EBI), Wellcome Genome Campus, Hinxton CB10 1SD, United Kingdom; European Molecular Biology Laboratory, European Bioinformatics Institute (EMBL-EBI), Wellcome Genome Campus, Hinxton CB10 1SD, United Kingdom; European Molecular Biology Laboratory, European Bioinformatics Institute (EMBL-EBI), Wellcome Genome Campus, Hinxton CB10 1SD, United Kingdom; Protein Information Resource, G eorgetown University Medical Center, 2115 Wisconsin Ave NW, G1 level, Suite 040A, Washington, DC 20007, United States; Protein Information Resource, U niversity of Delaware, Ammon-Pinizzotto Biopharmaceutical Innovation Building, Suite 147B, 590 Avenue 1743, Newark, DE 19713, United States; SIB Swiss Institute of Bioinformati cs, Centre Medical Universitaire, 1 rue Michel Servet, CH-1211 Geneva 4, Switzerland

## Abstract

The UniProt REST API is a freely available, open-access resource that powers the UniProt.org website and gives users flexible programmatic interaction with protein knowledge data. It provides access to UniProtKB, UniRef, UniParc, Proteomes, GeneCentric, ARBA, UniRule, and the ID Mapping tool, along with supporting data and controlled vocabularies. Users can access the API with their favorite programming language and generate example code snippets to access the UniProt databases using the API documentation page (https://www.uniprot.org/api-documentation) in various languages. API results can be returned and downloaded in various formats. With an average of 303 million requests per month over the last year, the API enables structured search queries using logical operators and parentheses, allows users to specify fields of interest within results, and customize downloads for direct integration into workflows. The API is a powerful tool that empowers users to fully utilize UniProt data across multiple datasets, enabling download, analysis, and extraction of valuable research insights. This website is free and open to all users, and there is no login requirement.

## Graphical Abstract

**Graphical Abstract:**
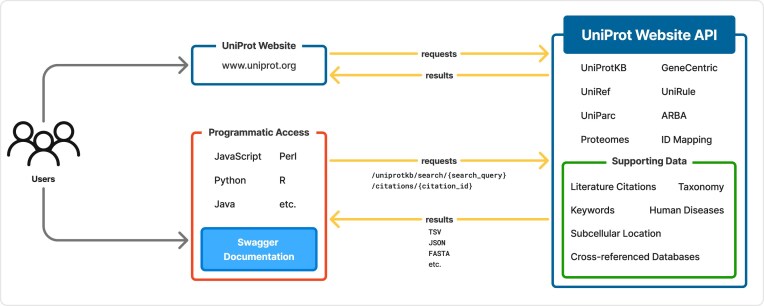

## Introduction

The ongoing worldwide proliferation of biological data has spurred the need for advanced data organization and accessibility portals for users. Researchers are presented with the challenge of integrating large amounts of complex data from diverse databases and formats to advance their research and contextualize their findings. Having free access to resources that collect, organize, and effectively present data from bioinformatics databases has become increasingly vital for scientific communities. This access helps to power research pipelines, provide context for new discoveries, and increase the overall impact of scientific findings.

The Universal Protein Resource (UniProt) [1] is a comprehensive suite of databases providing the scientific community with freely accessible, high-quality protein sequence and function information. This includes molecular functions, biological processes, cellular localization, molecular interactions, and disease-associated variations, along with detailed positional features such as active sites, binding sites, disulfide bonds, regions, and domains. UniProt integrates metadata from 180 cross-referenced databases spanning genomics, proteomics, protein function, and variation data, establishing a network of interconnected data resources and their experimental datasets. This integration enables researchers to conduct analyses across diverse biological data sources. In the UniProt Knowledgebase (UniProtKB), protein sequences are assigned stable identifiers and are enriched by manual and automatic annotation processes, summarizing protein functions and experimental evidence. The UniProt Reference Clusters (UniRef) database provides protein sequence clustering at various levels of sequence identity and the UniProt Archive (UniParc) maintains a complete, non-redundant repository of known protein sequences, including database cross-references and a comprehensive change history.

Here, we present the UniProt website API, which supports UniProt.org and provides programmatic access to protein knowledge across the UniProt databases. We provide an overview of the available data, outline the API’s architecture, demonstrate how to search and retrieve information, showcase illustrative examples, and conclude with a description of the help documentation.

## Data service descriptions

The website REST API provides access to all UniProt databases and tools through dedicated services, summarized in Table 1. Further details are available in the UniProt API documentation (https://www.uniprot.org/api-documentation), which is also discussed later in this paper. Each data service operates independently, providing access to its respective database. However, these services can be connected through shared data types and identifiers, enabling users to query and integrate data across multiple databases.

**Table 1.** Overview of databases with descriptions, use cases, and documentation links

Database	Description	Size (release 2025_01)	Use cases	Documentation endpoint URLs after https://www.uniprot.org/api-documentation
UniProtKB	Central database for protein sequence and function information that also provides non-redundant, stable identifiers for protein entries that can be used across all UniProt datasets.	253 206 170 protein entries consisting of 572 970 reviewed entries and 252 633 200 unreviewed entries	Search for protein function and sequence feature annotations from computational analysis and manual curation from the literature	/uniprotkb
UniRef	Clustered sets of sequences from UniProtKB and UniParc that are related based on sequence similarity at 100%, 90%, and 50%	UniRef100—453 950 711 clusters UniRef90—204 806 910 clusters UniRef50—69 290 910 clusters	Analyze protein clusters based on precomputed sequence similarity and coverage of the sequence space	/uniref
UniParc	A comprehensive, non-redundant protein sequence archive that provides stable, unique identifiers to sequences from public databases and contains a comprehensive list of all source datasets that contain that sequence and a record of the history of changes to that sequence	916 871 247 entries	Search across all protein sequences known to UniProt for sequence changes and cross-reference databases, including UniProtKB	/uniparc
Proteomes	Complete sets of proteins believed to be expressed by an organism that are identified by a unique proteome identifier	843 388 proteomes Consisting of 25 247 reference proteomes and 818 141 non-reference proteomes	Download complete sets of proteins for a user’s species of interest, which could include reviewed and unreviewed protein entries	/proteomes
GeneCentric	Groups entries for a set of gene products under a single gene identifier based on Ensembl, EnsemblGenomes, and model organism databases	83 086 888 UniProtKB entries	Retrieve protein products associated with a UniProtKB entry, proteome, or canonical gene identifier	/proteomes#GeneCentric
Supporting Data	Literature citations	2 038 892	Search publications that are computationally mapped or curated in UniProtKB protein entries	/support-data#Literature_citations
	Keywords	1201	Search for controlled vocabulary keywords that summarize the content of a protein entry	/support-data#Keywords
	Human disease	6740	Search for controlled vocabulary human diseases that are associated with UniProtKB	/support-data#Human_diseases
	Subcellular locations	564	Search for subcellular location compartment terms	/support-data#Subcellular_locations
	Cross-referenced databases	180	Search cross-referenced databases that are linked with UniProtKB protein entries	/support-data#Cross-referenced_databases
	Taxonomy	2 998 013	Search the UniProt taxonomy database	/support-data#Taxonomy
Automatic Annotation	Automatic classification and annotation using UniRule and ARBA systems to enrich entries with rule-based functional annotation	UniRule: 9398 ARBA: 36 645	Search automatic annotation rules for the list of conditions that are automatically applied by that rule, as well as the specific corresponding annotations	/aa
ID Mapping	Allows mapping of identifiers used in one database to identifiers used in another database		Map identifiers between UniProt and cross-referenced databases, and retrieve batches of UniProt entries using UniProt IDs	/idmapping

UniProtKB primary accessions serve as the central unifying keys. With a UniProtKB primary accession, users can retrieve detailed annotations and cross-references from UniProtKB, retrieve archived sequence versions in UniParc, explore sequence similarity clusters in UniRef, and identify the associated proteomes. Finally, the supporting data databases are utilized throughout UniProt, enhancing the consistency and depth of protein information available. The UniProt databases have been extensively detailed in a previous publication [1]. To facilitate seamless data integration, the ID Mapping service (https://www.uniprot.org/api-documentation/idmapping) allows users to map database identifiers (UniProt or cross-referenced) to another database. This enables users to establish links between biological databases for their protein of interest and enrich their result datasets with UniProtKB entry information [2].

## Architecture, design, and implementation

With the ever-growing size and complexity of biological data, it is essential for the underlying architecture to scale effectively while ensuring fast and efficient data retrieval to meet the needs of our users. To meet these challenges, our system is built with a modular design that incorporates several key components: API Gateway, service layer, search engine, and key-value store.

**Figure 1.**
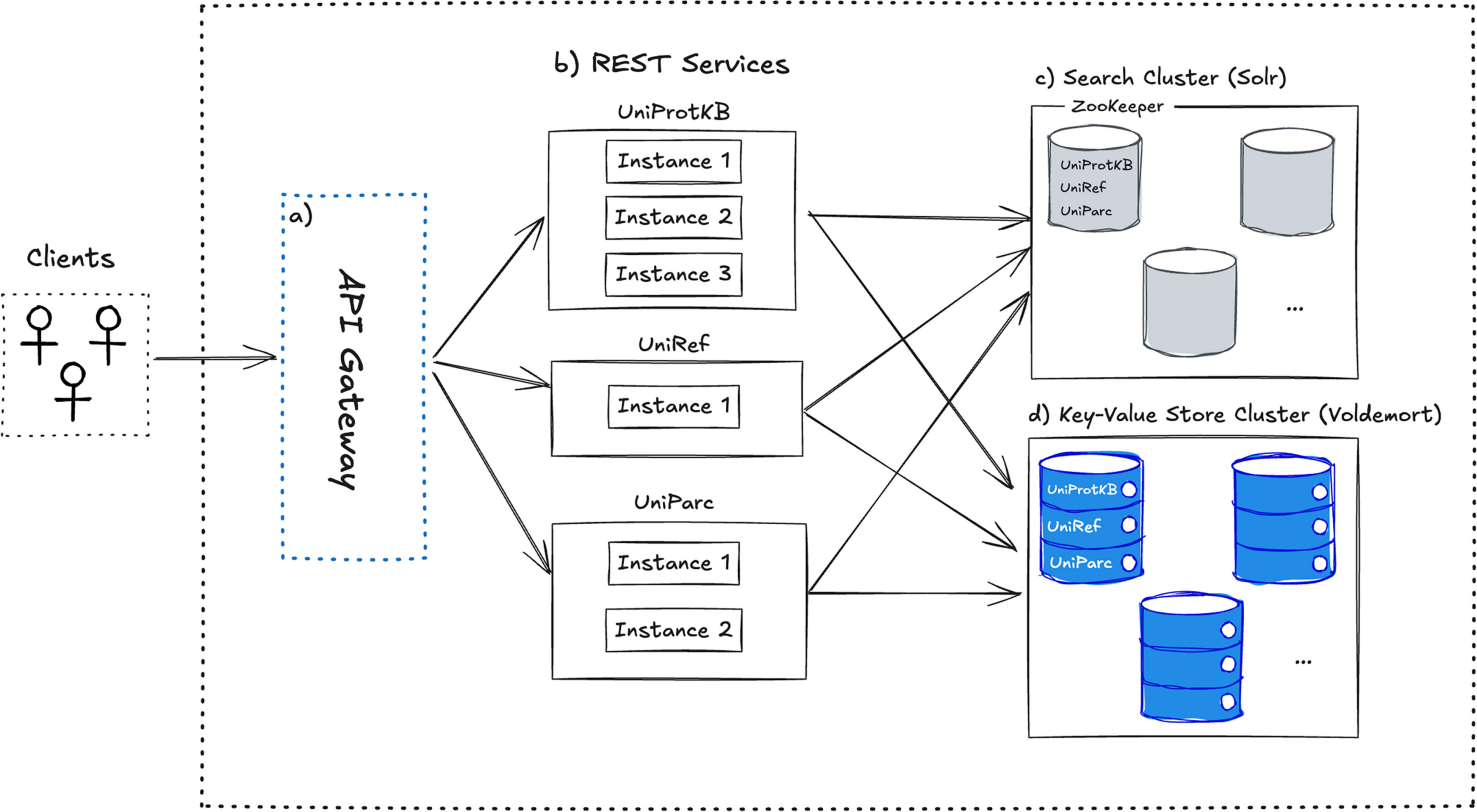
UniProt website API service architecture: This high-level architecture diagram shows a system where different clients (web, mobile, and programmatic) interact with back-end services via an API Gateway. (**a**) The API Gateway routes requests to (**b**) multiple REST services (UniProtKB, UniRef, UniParc, etc.), each running multiple instances for scalability and redundancy. These REST services retrieve data from two back-end storage systems: (**c**) Search Cluster (Solr)—handles querying of documents for fast search based on user’s search criteria. (**d**) Key-Value Store Cluster (Voldemort)—provides efficient, low-latency storage for key-value-based lookups.

The API Gateway (Fig. 1a) is the entry point for all user requests (programmatic clients, web browsers, or bots) and routes them to the appropriate service, such as UniProtKB, UniRef, or UniParc (Fig. 1b). The number of instances for each service can be auto-scaled depending on user demand. These services are implemented in Java using Spring Boot (spring.io/projects/spring-boot).

Each service within the UniProt website API interacts with two distributed systems: Solr (Fig. 1c) (solr.apache.org) for search operations and Voldemort (Fig. 1d) (github.com/voldemort/voldemort) for data retrieval. The use of distributed systems enhances scalability, performance, and fault tolerance, ensuring that the API can efficiently handle large-scale queries and data requests. To maintain system reliability, we manage our Solr clusters using ZooKeeper (zookeeper.apache.org), which coordinates servers and manages configuration settings.

Solr processes user queries through its high-performance search engine, returning matching IDs. These IDs serve as references to retrieve complete UniProt entries from Voldemort, which stores the full annotated data, including biological information, functional annotations, and cross-references. Voldemort is designed for low-latency access and supports horizontal scaling with in-memory caching to further optimize performance. To reduce retrieval times, all data in Voldemort are compressed using the Brotli algorithm (github.com/google/brotli), reducing the size of stored and transmitted data by 14% and lowering the latency by 20% when compared with typical gzip compression.

## Data search and retrieval

The UniProt website API allows RESTful access to the various UniProt databases. Each database has three common types of endpoints: entry, search, and stream (see Table 2). They support multiple response formats, including tabular forms like TSV or Excel, or more complex structures like JSON, XML, RDF, or FASTA format (see https://www.uniprot.org/api-documentation). This variety of popular formats allows for easier integration into diverse user workflows without requiring additional transformation on the user’s side.

**Table 2.** Common API endpoints, structures, example requests, and use cases

Endpoint name	Structure	Example URLs after https://rest.uniprot.org	Typical use cases	Customization
Entry	https://{host}/{database}/{id}	https://rest.uniprot.org/uniprotkb/P05067 https://rest.uniprot.org/uniref/UniRef100_P05067 https://rest.uniprot.org/uniparc/UPI000002DB1C	Download one single entry from its accession Download some (<50) entries from their accessions through multiple requests To download more entries from their accessions, ID Mapping would be recommended instead	Field filtering Format selection
Search	https://{host}/{database}/ search?query={search query}	https://rest.uniprot.org/uniprotkb/search?query=gene:APP https://rest.uniprot.org/uniref/search?query=name:APP https://rest.uniprot.org/uniparc/search?query=gene:APP	Download a paginated result set from a query, by pages of up to 500 results No limit to the number of individual requests Requires additional logic to loop through pages of results, compressed or uncompressed	Field filtering Field sorting Format selection
Stream	https://{host}/{database}/ stream?query={search query}	https://rest.uniprot.org/uniprotkb/stream?query=gene:APP https://rest.uniprot.org/uniref/stream?query=name:APP https://rest.uniprot.org/uniparc/stream?query=gene:APP	Download a full result set from a query, in one streamed request A single request can include up to 10 million results Avoids having to write logic to loop through all the result pages, but the download is not resumable: if there is any connection issue during the download, the whole download needs to be restarted	Field filtering Format selection

Code examples and further information are also provided in the documentation: https://www.uniprot.org/help/api_queries.

The *entry* endpoint retrieves a specific entry using its unique identifier. The *search* endpoint returns a paginated list of entries matching a given query, which can be either a plain text search or an advanced query with specific fields. Pagination is crucial for efficiently displaying results, preventing the system from loading millions of entries at once. The *stream* endpoint accepts the same input as the search endpoint but returns all results in a single response, enabling large-scale retrieval. However, this approach has the trade-off that the request cannot be resumed if the connection is lost.

As mentioned earlier, the search endpoint is a paginated API. It returns a page of results and a “Link” response header to fetch the next page if it exists. By default the page size is 25, which can be changed by passing the request query parameter “size” with a maximum page size of 500. In contrast, the stream API downloads all the results at once with a limit of 10 million on the result size either in compressed or uncompressed formats.

There are common query parameters in each endpoint. For example, there is the “fields” parameter to request a list of comma-separated values in the response (e.g. fields = accession,organism_name,gene_names). For tabular formats such as TSV, fields are returned in the requested order, enabling granular customization of the response and simplifying downstream usage in a user workflow.

The *search* and *stream* endpoints have a common query parameter called “query” across the different databases. The “query” parameter is used to pass the search criteria, e.g. “query = protein_name:rubisco.” The search criteria can be either free text or a valid Solr query with logical operators AND, OR, and NOT. For the search endpoints, the “sort” parameter allows the user to sort the result on a certain field or list of fields [e.g. query = “((gene:CTNNB1) AND (taxonomy_id:9606))” and sort = organism_name asc].

In each of the endpoints, we have included additional HTTP response headers that users might be interested in, such as total number of query hits (X-Total-Results), open next page of results (Link), release version (X-UniProt-Release), and release date (X-UniProt-Release-Date).

In addition to the common endpoints, we have light endpoints for UniParc and UniRef to get the core data of an entry. UniRef has the /members endpoint to obtain cluster members for a given cluster ID. We also provide two additional endpoints in UniParc to retrieve cross-references by UniParc ID: /uniparc/{upi}/databases for paginated results and /uniparc/{upi}/databases/stream for downloading all cross-references. All of our endpoints have validations and error handling with appropriate HTTP status codes (see Supplementary Table S1).

The underlying data for the UniProt website are updated every 8 weeks, with the code updated more frequently with new features and improvements. The website API is not linked to the data release, which makes it quicker to release fixes and improvements. We announce upcoming major changes one release in advance, allowing users to prepare accordingly; all previous changes are documented in the release notes (https://www.uniprot.org/release-notes?query=*).

### ID Mapping service

The ID Mapping service is an asynchronous job API service to map the identifiers of one database to another [2], e.g. RefSeq IDs to UniProtKB IDs. ID Mapping has four main API endpoints that allow the user to submit a job, obtain the status, and download the results (see Table 3).

**Table 3.** ID Mapping service API calls

Name of the endpoint	Endpoint URL after https://rest.uniprot.org
Submit an ID Mapping job	/idmapping/run
Get the status of a job	/idmapping/status/{jobld}
Download results—paginated	/idmapping/results/{jobId}
Download results—all	/idmapping/stream/{jobId}

Users are able to download the mapped ID results with UniProt data if the target mapping is to UniProtKB, UniRef, or UniParc entries. In the case of mapping from UniProt IDs to third-party databases, the user will receive only the database ID, allowing them to use these other database APIs or services to retrieve further data not directly related to UniProt.

Similar to other endpoints, users are able to obtain either paginated results that can be queried and sorted or all results at once through the last two endpoints in Table 3. Please refer to the UniProt REST tutorial (https://www.uniprot.org/help/uniprot_rest_tutorial) for an example user case. A user may submit multiple ID Mapping jobs; however, a single job request is limited to a maximum of 100 000 IDs. Job results are stored on the server for a maximum of 7 days, after which jobs will need to be resubmitted.

## Example use cases

For detailed programmatic methodology of both example use cases, please see the Supplementary data. Results and methodology are correct as of release 2025_01.

### Use case 1: cross-database querying using the website API

The Website API provides an intuitive and versatile way for users to access the knowledge stored in the UniProt datasets and integrate it into their own analysis pipelines. To showcase the ability of the website API to search across UniProt datasets, we reference the sequence archive UniParc which provides links to UniProtKB, a rich, interconnected database of protein function and feature annotation. Additionally, as an example of how to use data from cross-referenced databases to search UniProt datasets, we will be using a nasal swab sequence stored in the European Nucleotide Archive (ENA) of which we assume the user does not have prior knowledge of the proteins’ identity.

The user is provided with a sequence of a SARS-CoV-2 isolate (which was sequenced from a nasal swab in Washington in March 2020) and wishes to use it to understand the mechanism of SARS-CoV-2 transmission to wild white-tailed deer during the global pandemic [3, 4].

The sequence was originally submitted to ENA with a unique identifier QIZ14413 (https://www.ebi.ac.uk/ena/browser/view/QIZ14413). A search within UniProtKB for this identifier returns no results (https://www.uniprot.org/uniprotkb?query=QIZ14413). As UniParc (www.uniprot.org/help/uniparc) is a database of protein sequences sourced from public sequence databases, including ENA, it can be searched via source database identifiers (such as QIZ14413 (https://www.uniprot.org/uniparc?query=QIZ14413)) which will return the unique protein sequence stored with a stable unique identifier, in this case UniParc ID UPI00131F240A (https://www.uniprot.org/uniparc/UPI00131F240A/entry). Within this entry’s cross-references, we see P0DTC2 (https://www.uniprot.org/uniprotkb/P0DTC2/entry), which is a reviewed UniProtKB entry specifically for SARS-CoV-2 spike glycoprotein containing a wealth of protein function and feature annotations (https://www.uniprot.org/uniprotkb).

Spike glycoprotein (P0DTC2) is a structural protein of SARS-CoV-2 that mediates virus entry into the host cell [5]. To determine which host protein spike glycoproteins bind to, manually curated interaction data can be identified by extracting subunit structure data from the P0DTC2 entry (https://www.uniprot.org/uniprotkb/P0DTC2/entry#interaction). This returns evidence from publications that indicate interactions with human angiotensin-converting enzyme 2 (hereafter referred to as ACE2) [6] and a search of UniProtKB for reviewed entries with the gene name ACE2 in human finds the entry Q9BYF1 (https://www.uniprot.org/uniprotkb/Q9BYF1/entry).

Querying for “regions” in ACE2 sequence features identifies three distinct regions that are annotated as binding to SARS-CoV spike glycoprotein: regions between the residue numbers 30–41, 82–84, and 353–357 (https://www.uniprot.org/uniprotkb/Q9BYF1/entry#family_and_domains). Examining the mutagen features at these residue ranges confirms there is experimental evidence for SARS-CoV-2 spike glycoprotein interaction at these sites, suggesting that these regions could be fundamental in the interaction with both SARS-CoV and SARS-CoV-2 spike glycoproteins (https://www.uniprot.org/uniprotkb/Q9BYF1/entry#disease_variants).

To determine whether these regions of interaction between human ACE2 and spike glycoproteins are conserved in white-tailed deer (TaxID: 9874), a search for the ACE2 gene in white-tailed deer in UniProtKB returns the entry A0A6J0Z472 (https://www.uniprot.org/uniprotkb/A0A6J0Z472/entry). Alignment of A0A6J0Z472 and Q9BYF1 using the EBI’s Clustal Omega Multiple Sequence Alignment service (https://europepmc.org/article/MED/38597606) indicates reasonable evolutionary conservation between these species. Focusing on the spike glycoprotein interaction regions indicated in human ACE2 indicates that all three of these regions are highly conserved in white-tailed deer. This suggests that the infection initiation mechanism in humans of spike glycoprotein binding to host ACE2 is also likely to be the mechanism by which SARS-CoV-2 is able to infect white-tailed deer [7].

### Use case 2: ID Mapping service

The ID Mapping service (https://www.uniprot.org/id-mapping) allows users to map identifiers from one database to another database. This is a vital feature when using multiple source databases or processing software as part of a larger integrated analysis pipeline. Conversion of gene names to database identifiers is commonly used to facilitate further analysis and understanding of a results dataset. In this example, gene names are entered as the input and UniProtKB/Swiss-Prot IDs as the output with a taxonomy restriction (e.g. human TaxID: 9606) to restrict the results to species-specific identifiers. Results identify a one-to-one identifier mapping. Additionally, users can download the protein entry information from the output UniProtKB entries.

### API documentation and web interface

We provide REST API documentation through Swagger (https://www.uniprot.org/api-documentation), which follows the OpenAPI specification 3.x. Swagger enables users to explore API endpoints, test API calls interactively, and generate request snippets in various languages, including Bash (cURL), Python, JavaScript, Perl, and R. Users can navigate through different tabs to find the appropriate API for their dataset.

We provide a React-based web interface (https://www.uniprot.org/) for searching and visualizing dataset entries. As a thin client, it relies on our REST API to dynamically populate the page. The web interface groups search results by various attributes, making filtering easier and helping with the exploration of the API capabilities by exposing them through the User Interface (UI). It can also generate REST API URLs based on user queries that can then be shared with other users. Additionally, users can download search results in supported formats. Beyond search functionality, the web interface serves as a valuable resource for biological knowledge, help documentation, upcoming changes, and online training advertisements.

## Discussion

The primary goal of our REST APIs is to provide platform-independent access to comprehensive, high-quality protein-related data available in the UniProt databases. Users can query and retrieve data in multiple formats, enabling the scientific community to access information according to their specific research needs. Our extensive Swagger documentation details the full range of data attributes and access methods, with practical examples provided across multiple programming languages. The APIs are designed for seamless integration, allowing researchers to automate data retrieval, conduct large-scale analyses, and efficiently incorporate UniProt data into their workflows. Our APIs are free to use and do not require login or authentication, ensuring open and easy access for all users.

## Supplementary Material

gkaf394_Supplemental_File

## Data Availability

API is freely available at https://rest.uniprot.org/. API documentation is freely available at https://www.uniprot.org/api-documentation/.

## UniProt Consortium members’ list

Alex Bateman, Maria-Jesus Martin, Sandra Orchard, Michele Magrane, Shiqi Ye, Aduragbemi Adesina, Shadab Ahmad, Emily H. Bowler-Barnett, David Carpentier, Paul Denny, Jun Fan, Leonardo Jose da Costa Gonzales, Abdulrahman Hussein, Alexandr Ignatchenko, Giuseppe Insana, Rizwan Ishtiaq, Vishal Joshi, Dushyanth Jyothi, Swaathi Kandasaamy, Antonia Lock, Aurelien Luciani, Jie Luo, Yvonne Lussi, Juan Sebastian Martinez Marin, Pedro Raposo, Daniel L. Rice, James Stephenson, Prabhat Totoo, Nadya Urakova, Preethi Vasudev, Supun Wijerathne, Khawaja Talal Ibrahim, Minjoon Kim and Conny Wing-Heng Yu at the EMBL-European Bioinformatics Institute; Alan J. Bridge, Lucila Aimo, Ghislaine Argoud-Puy, Andrea H. Auchincloss, Kristian B. Axelsen, Parit Bansal, Delphine Baratin, Teresa M. Batista Neto, Marie-Claude Blatter, Jerven T. Bolleman, Emmanuel Boutet, Lionel Breuza, Cristina Casals-Casas, Kamal Chikh Echioukh, Elisabeth Coudert, Beatrice Cuche, Edouard de Castro, Anne Estreicher, Maria L. Famiglietti, Marc Feuermann, Elisabeth Gasteiger, Pascale Gaudet, Sebastien Gehant, Vivienne Gerritsen, Arnaud Gos, Nadine Gruaz, Chantal Hulo, Nevila Hyka-Nouspikel, Florence Jungo, Arnaud Kerhornou, Philippe Le Mercier, Damien Lieberherr, Patrick Masson, Anne Morgat, Salvo Paesano, Ivo Pedruzzi, Sandrine Pilbout, Lucille Pourcel, Sylvain Poux, Monica Pozzato, Manuela Pruess, Nicole Redaschi, Catherine Rivoire, Christian J.A. Sigrist, Karin Sonesson, Shyamala Sundaram and Anastasia Sveshnikova at the SIB Swiss Institute of Bioinformatics; Cathy H. Wu, Chuming Chen,, Hongzhan Huang, Kati Laiho, Minna Lehvaslaiho, Peter McGarvey, Darren A. Natale, Karen Ross, C.R. Vinayaka, Yuqi Wang, Raja Mazumber, Vijay Shanker, and Jian Zhang at the Protein Information Resource.
