# Supplementary material for: The UniProt website API: facilitating programmatic access to protein knowledge
Source: Nucleic Acids Res. 2025 May 7;53(W1):W547–53. doi: 10.1093/nar/gkaf394 (PMC12230682; doi:10.1093/nar/gkaf394)
Supplement: gkaf394_Supplemental_File [file gkaf394_supplemental_file.pdf]

MANUSCRIPT TITLE

The UniProt website API: facilitating programmatic access to protein knowledge.

SUPPLEMENTARY DATA

Supplementary table 1

HTTP status code	Meaning
2xx	Request is successful
3xx	Redirection + redirect URL
4xx	Bad request + relevant error messages to facilitate corrective action
429	IP rate limit reached
5xx	Issue with our services

Supplementary Table 1 - Error HTTP status codes and their meaning, these are provided for all of our endpoints.
